# Vitamin D status in Saudi school children based on knowledge

**DOI:** 10.1186/s12887-015-0369-9

**Published:** 2015-05-06

**Authors:** Yousef Al-Saleh, Nasser M Al-Daghri, Nasiruddin Khan, Hanan Alfawaz, Abdulaziz M Al-Othman, Majed S Alokail, George P Chrousos

**Affiliations:** King Abdulaziz Medical City, College of Medicine, King Saud Bin Abdulaziz University for Health Sciences, Riyadh, 11426 Saudi Arabia; Prince Mutaib Bin Abdullah Chair on Osteoporosis, Biochemistry Department, College of Science, King Saud University, PO Box, 2455, Riyadh, 11451 Kingdom of Saudi Arabia; Biomarkers Research Program Biochemistry Department, College of Science, King Saud University, Riyadh, Saudi Arabia; Department of Food Science and Nutrition, College of Food Science and Agriculture, King Saud University, Riyadh, Saudi Arabia; College of Applied Medical Sciences, King Saud University, Riyadh, Saudi Arabia; First Department of Pediatrics, Athens University Medical School, Athens, 11527 Greece

**Keywords:** Hypovitaminosis D, Awareness, Sunlight, Physicians, Teacher, Gender

## Abstract

**Background:**

The prevalence of vitamin D deficiency in the kingdom of Saudi Arabia is rising unexpectedly in every age group. Apart from several risk factors, the lack of awareness is posing a serious threat for low vitamin D levels in children as well. The aim of our study was to compare the knowledge and status of vitamin D in Saudi school children.

**Methods:**

Saudi students, 1188 boys (15.1 ± 2.2 years) and 1038 girls (15.1 ± 2.0 years), were recruited and a pre-designed questionnaire with regards to knowledge about vitamin D was administered. Blood samples were collected and serum 25hydroxyvitamin D (25(OH)D was measured.

**Results:**

A significantly higher percentage of boys answered correctly than girls regarding knowledge questions as sun exposure (p = 0.002, and 0.011), breastfeeding (p < 0.001) and diseases (p < 0.001). The percentage of girls was significantly higher who thought that fruits and vegetables are not rich sources of vitamin D (24.7% girls vs. 15.4% boys; p < 0.001and 29.6% girls vs. 20.9% boys p < 0.001), respectively. Boys had a higher prevalence and frequency of sun exposure than girls (p < 0.001 for both). Girls showed a significantly higher percentage of sunscreen use and full covering during sun exposure (p = 0.001 for both).Vitamin D deficiency was significantly higher in girls than boys (47.0% versus 19.4.0%; p < 0.001). Vitamin D status in boys was significantly higher than girls (p < 0.001). In girls, those who answered correctly about vitamin D related disease (p = 0.03) and sources (p = 0.015), demonstrated significantly higher vitamin D levels.

**Conclusions:**

The awareness of vitamin D and sunlight in children needs to be improved by provision of trained physicians and school teachers. Creating more areas where girls can uncover freely during routine works and outdoor activities will help increase their vitamin D levels.

## Background

The worldwide increased prevalence of vitamin D deficiency in both children and adults [1] has been related to various factors, including exposure time of skin to ultraviolet radiation [2], skin pigmentation and vitamin D intake [3]. Moreover, vitamin D receptor (VDR) polymorphisms [4], obesity and social status [5-7] could also play important roles in modulating the levels of circulating vitamin D. Despite sufficient sunlight throughout the year, the kingdom of Saudi Arabia (KSA) is facing a common problem of vitamin D deficiency in both children and adults [8-10].

A recent study amongst Saudi locals and expatriates revealed higher vitamin D levels during winter months than in the summer [11]. The reason for this unexpected trend in the summer was the avoidance of sunlight to prevent from scorching heat effects on skin and health. In addition, girls are more vitamin D deficient as they receive little or no sunlight due to the covering of their bodies with dark veils for cultural and religious reasons. A recent study from our group in 331 Saudi children aged 6–17 years (153 boys and 178 girls) demonstrated that all subjects were vitamin D deficient (serum 25-(OH) vitamin D) with the majority being moderately deficient (71.6%) [12].

It is now becoming clear that apart from several risk factors, the lack of awareness is also posing a serious threat for low vitamin D levels almost in every age group. For instance, the Australian surveys showed a limited awareness and understanding about vitamin D in the adults [13]. There are studies from different parts of Saudi Arabia demonstrating that a limited knowledge and awareness about vitamin D could be a potential reason for vitamin D deficiency among female students [14,15]. Hence, the understanding and knowledge of the importance and role of vitamin D in one’s normal daily life is a basic need and the general community, especially children, should be aware of this.

There are several studies from Saudi Arabia regarding knowledge about vitamin D and its deficiency. However, these either included adults or only female students as participants. On the contrary, this study focused on school children, both boys and girls, comparing their knowledge about the role and importance of vitamin D and its deficiency. In addition, the correlation of such knowledge and vitamin D status in school children was also examined.

## Methods

### Subjects and data collection

A total of 2226 apparently healthy Saudi students were recruited from randomly selected public secondary schools within Riyadh, Saudi Arabia, and consisted of 1188 boys (mean age, 15.1 ± 2.2 years) and 1038 girls (15.1 ± 2.0). Written informed consents from the parents of adolescents were obtained prior to inclusion. Subjects with chronic conditions such as asthma and type 1 diabetes mellitus, those needing acute medical attention, use medications known to affect body weight (such as steroids) and those taking calcium and multivitamin supplements, were excluded from the study. Ethical approval was obtained from the Ethics Committee of the College of Science Research Center, King Saud University, Riyadh, Saudi Arabia. Approval to conduct the study and access to students were also obtained from the Ministry of Education. A pre-designed and approved questionnaire, which included information on socio-demographics, polar questions (yes or no questions) with regards to knowledge about vitamin D and vitamin D-associated diseases were administered to all subjects along with multiple choice questions about sunshine exposure and duration.

### Anthropometrics

Physical examination was carried out by the school attending physician and nurse during break time to avoid class disruption. Weight and height were recorded to the nearest 0.2 kg and 0.5 cm, respectively, using an appropriate international standard scale (Digital Pearson Scale, ADAM Equipment Inc., USA). Waist circumferences were measured using non-stretchable tape. Definition of BMI was based on the cutoffs proposed by Cole and colleagues [16].

### Vitamin D status analysis

Blood samples were collected by the school physician and nurse immediately after anthropometrics. Serum 25(OH)D was measured using COBAS e-411 automated analyzer (Roche Diagnostics, Indianapolis, IN, USA) in a DEQAS (Vitamin D External Quality Assessment Scheme) accredited laboratory (Prince Mutaib Chair for Biomarkers of Osteoporosis, Biochemistry Department, College of Science, King Saud University, Riyadh, Saudi Arabia). The various states of vitamin D status were defined as: deficient (<25 nmol/L); insufficient (25-50 nmol/L); sufficient (50-75 nmol/L) and desirable (>75 nmol/L). Subjects diagnosed with vitamin D deficiency were subsequently referred to the school physician for further guidance.

### Statistical analysis

Data was analyzed using SPSS version 16.0 (SPSS, Chicago, IL, USA). Frequencies were presented as percentages (%) and continuous variables were presented as mean ± standard deviation. Chi-Square test was done to compare frequencies and independent Student-test for normally distributed continuous variables. Significance was set at p-value < 0.05.

## Results

### Vitamin D awareness

Table 1 highlights the demographics of boys and girls, as well as their knowledge on vitamin D and vitamin D-related diseases and sun exposure. There is a significant difference in skin color distribution among subjects, with girls having a higher prevalence of “fair-toned” skin than boys (p <0.001). Housing-wise, the majority of the students lived in villas, with girls having a modestly higher prevalence of villa residency than boys (p < 0.001). With regards to vitamin D knowledge, there was a significantly higher percentage of boys answering “yes” than girls in 5 out of 12 questions, 4 of which was correct [30 minute sun exposure provides enough calcium and vitamin D, about breastfeeding, and vitamin D deficiency leads to rickets in children; p-values 0.002, 0.011 0.001, and 0.001 respectively]. On the other hand, while the majority of both boys and girls thought that fruits and vegetables are rich in vitamin D, a significantly higher percentage of girls got it correct (24.7% girls versus 15.4% boys; p-value <0.001 and 29.6% girls versus 20.9% boys; p-values <0.001, not shown in table). Worthy to note is the low percentage of both boys and girls having heard of vitamin D sources in both schools and primary care centers, which means that knowledge on vitamin D and its sources were probably acquired elsewhere (e.g. parents, media). As for sun exposure, boys had a higher prevalence of sun exposure than girls as well as increased frequency (p-values 0.001, for both). Consequently and as expected, girls had a significantly higher percentage of sunscreen use and full covering during sun exposure (p-values 0.001 and 0.02, for both) (Table 1).

**Table 1:** **Demographics and vitamin D awareness of subjects**

**Parameters**	**Boys**	**Girls**	**P-value**
N	1188	1038	
Age (years)	15.1 ± 2.2	15.1 ± 2.0	NS
Skin Color			
Fair	31.4	39.1	< 0.001
Light Brown	60.1	56.4	
Dark Brown	7.8	4.4	
Black	0.7	0.1	
Housing			
Apartment	33.5	25.2	<0.001
Villa	63.4	69.3	
Public House	3.0	5.5	
Education			
Elementary	5.9	2.9	NS
Primary	55.8	54.5	
Secondary	38.1	42.3	
College/Post-Graduate	0.3	0.3	
Do you take vitamin D supplements?	45.7	41.9	NS
Vitamin D Knowledge*			
Have you heard about vitamin D sources in school?	45.7	47.9	NS
Have you heard about vitamin D sources in primary care centers?	27.5	32.1	0.018
Does 30 min exposure to sun provide enough calcium?	63.9	57.8	0.002
Does 30 min exposure to sun provide enough vitamin D?	76.5	72.1	0.011
Are vegetables rich in vitamin D?	79.1	70.4	<0.001
Are fruits rich in vitamin D?	84.6	75.3	<0.001
Does breastfeeding contain enough vitamin D?	81.5	71.7	<0.001
Is vitamin D important in calcium absorption?	84.4	83.8	NS
Does vitamin D deficiency lead to rickets in children?	90.9	85.6	< 0.001
Does vitamin D deficiency lead to osteomalacia?	85.3	84.1	NS
Does vitamin D deficiency lead to osteoporosis?	91.4	89.7	NS
What is the recommended dose for vitamin D?			
40-80 IU	42.0	41.6	0.016
400-800 IU	51.7	55.0	
4000-8000 IU	6.4	3.4	
Sunlight Exposure			
Do you expose yourself to sunlight?	77.5	54.3	<0.001
Frequency of sunlight exposure			
One to three times daily	55.7	41.2	<0.001
One to two times per week	31.3	33.2	
Three to five times per week	13.1	25.6	
Time of sunlight exposure			
Sunrise to 10 am	37.3	38.1	NS
10-am-3 pm	35.6	35.7	
After 3 pm	27.2	26.2	
Do you expose yourself to sunlight all-year through?	62.9	41.8	<0.001
Do you expose yourself to sunlight on certain seasons?	38.5	45.1	0.001
Are you fully covered during sun exposure?	22.5	28.3	0.001
Do you use sunscreen?	3.0	7.9	0.001

*Percentage of all responses corresponds to “YES”, NS: non-significant.

### Vitamin D status

Vitamin D deficiency was significantly higher in girls than boys (47.0% versus 19.4%; p < 0.001), with less than 3% of the entire cohort achieving desirable vitamin D levels and majority falling under the vitamin D insufficient category (Table 2). Accordingly, serum mean vitamin D level in boys was significantly higher than girls (39.0 ± 0.6 versus 29.5 ± 0.6 nmol/l, respectively).

**Table 2:** **Biochemical characteristics of the subjects (children)**

**Parameter**	**Boys**	**Girls**	**P-value**
N	1188	1038	
Vitamin D Status			
Deficient (%)	19.4	47.0	<0.001
Insufficient (%)	59.6	45.5	
Sufficient (%)	17.2	4.7	
Desirable (%)	3.9	2.7	
BMI (kg/m^2^)	22.9 ± 0.2	23.0 ± 0.2	NS
25-OH Vitamin D (nmol/l)	39.0 ± 0.6	29.5 ± 0.6	<0.001

Note: Data presented as percentage (%) for frequencies; mean ± standard error for continuous variables; *p*-value significant at < 0.05, NS: non-significant.

### Mean vitamin D levels and vitamin D knowledge

Vitamin D status was compared in 8 out of the 12 polar questions relating to vitamin D knowledge (Table 3). Both boys and girls who answered “Yes” to the question “Have you heard about vitamin D sources in primary care centers ?” have a significantly higher vitamin D levels as compared to “No” (p-values 0.03 and 0.05, respectively). In girls, mean vitamin D levels were significantly higher in those who answered “No” as to “whether vegetables are rich in vitamin D” (p-values 0.015) as compared to those who responded “Yes”. Similarly, significantly higher mean vitamin D levels (p-values 0.03) were observed in girls responding correctly to “Does vitamin D lead to osteoporosis” as compared to “No”.

**Table 3:** **Mean vitamin D levels based on general knowledge about vitamin D among schoolboys and girls**

	**BOYS**			**GIRLS**		
	**Yes**	**No**	**P-value**	**Yes**	**No**	**P-value**
Have you heard about vitamin D in school?	39.3 ± 0.9	38.1 ± 0.9	NS	29.8 ± 0.9	29.2 ± 0.7	NS
Have you heard about vitamin D sources in primary care centers	41.1 ± 1.6	37.4 ± 0.6	0.03	31.3 ± 1.2	28.7 ± 0.7	0.05
Is vitamin D important in calcium absorption?	39.1 ± 0.6	38.5 ± 1.2	NS	29.6 ± 0.6	28.9 ± 1.6	NS
Does vitamin D deficiency lead to rickets in children?	38.8 ± 0.6	40.2 ± 1.6	NS	29.7 ± 0.6	28.4 ± 1.1	NS
Does vitamin D deficiency lead to osteomalacia in adults?	38.8 ± 0.6	40.1 ± 1.6	NS	29.6 ± 0.6	29.3 ± 1.2	NS
Does vitamin D lead to osteoporosis?	38.8 ± 0.6	42.2 ± 2.2	NS	29.8 ± 0.6	26.1 ± 1.1	0.03
Are vegetables rich in vitamin D?	38.4 ± 0.6	40.3 ± 1.6	NS	28.6 ± 0.6	31.7 ± 1.2	0.015
Are fruits rich in vitamin D?	39.2 ± 0.6	38.3 ± 1.2	NS	29.2 ± 0.7	30.4 ± 1.2	NS

Note: Adjusted for the following confounding variables: age, obesity, skin color, use of sunscreen, use of vitamin D supplements, outdoor school exercise; p-value significant at <0.05, NS: non-significant.

## Discussion

The present study assessed vitamin D status in Saudi children searching for a possible correlation between vitamin D status and knowledge. We observed a significantly higher vitamin D deficiency in girls as compared to boys supporting various studies [13-15].

### Vitamin D awareness

This study demonstrates the lack of basic knowledge about vitamin D which was found to be more prevalent among girls than boys. Boys responded with more correct answers than girls (5 out of 12 questions, 4 of which are correct) regarding vitamin D. Gaps in basic knowledge around vitamin D and its benefits became apparent in this study. As described earlier, Christie et al. [14] demonstrated limited knowledge of female students (20 and 25 years) about vitamin D and vitamin D deficiency. In addition, Siddiqui et al. also showed a lack of awareness about the role of vitamin D among school girls (12–15 years) [15]. Our study supported the findings of Siddiqui et al. showing more lack of knowledge in girls. The lack of basic knowledge about sources of vitamin D in both boys and girls was also observed as they responded “yes” for the question “Are fruits rich in vitamin D?” which was incorrect. However, the percentage of girls (24.8%) was significantly higher than boys (15%) who gave correct answer responding “No”. The above results (Table 1) also correspond to the results described in Table 3 in which the girls responding correctly (No instead of yes) in the question “Are fruits rich in vitamin D?” demonstrated higher serum 25-OH vitamin D level than boys.

### Sources of information about vitamin D

Principally, in children, the primary source of basic knowledge about healthy diet (including minerals and vitamins) is obtained either from the schools or from primary care centers, although this was not the case in our study. The majority of the participants got the information about vitamin D from other sources like their parents and media. There are studies demonstrating the lack of nutritional knowledge in health care professionals from different countries rating from very poor to weak [17,18]. A recent study from United Arab Emirates demonstrated gaps in the knowledge as well as lack of management skills of physicians regarding bone health [19]. Another study from Qatar performed by Daradkeh et al. rated the nutritional knowledge in physicians as: very good (19%), moderate (60%), and poor (21%), indicating the need for nutrition to be included as an essential part for medical education [20].

Indeed, the few studies that have looked at vitamin D knowledge have found staggeringly low rates of awareness [14,21,22]. For example, a qualitative study conducted in Saudi Arabia found only minimal awareness of vitamin D knowledge, including where it comes from and what it does for health [14]. According to Khalsa, many health-care professionals are not fully aware of the benefits of vitamin D to public health [23]. Al-Numair et al. demonstrated that in Saudi Arabia, 75% of the physicians described their knowledge of nutrition as “poor” [24]. In addition, the majority of the physicians (81%) working in the Ministry of Health (MOH) primary health care centers in Jeddah, Saudi Arabia, rated their nutritional knowledge as “poor” based on their responses in a validated questionnaire [25].

### Sunlight exposure

The lack of sun exposure is among one of the main causes for vitamin D deficiency intake [26,27] in addition to prolonged breastfeeding and lack of a balanced diet [28,29]. Indeed, 80-100% of vitamin D requirement is fulfilled by exposure of the skin to sunlight [27]. In most parts of the Middle East, including Saudi Arabia, an indoor lifestyle has played a major role as an obstacle in obtaining sufficient amount of direct sun light, both in children and adults [30]. A recent study of school children from Makkah (KSA), observed higher vitamin D deficiency in females than males and reported the restriction of sunlight as the main cause [31]. In this study, the frequency of sunlight exposure was significantly higher in boys than girls. In Saudi Arabia, boys have more opportunity to spend most of their time outside their houses and are thus more exposed to sunlight than girls. On the other hand, girls have only few opportunities to go outside and uncover their faces or their bodies and expose themselves to the sun. The preferred outing time in the parks and malls for girls are usually in the evening, after sunset. In addition, the schools and transporting vehicles especially meant for girls are completely covered and prevent the penetration of proper sunlight.

Moreover, based on cultural traditions, concealing all of the body is considered another reason for insufficient sunlight as adapted by most Middle Eastern countries. Arab women who cover most of their skin are often vitamin D deficient [32]. The study by Siddiqui and colleagues demonstrated that women covering their body completely from head to toe limit severely sunlight exposure [15,33].

Similarly, severe vitamin D deficiency was also demonstrated in Arab–American women who wear the veil [34]. However, Sedrani and colleagues reported that, although veils could minimize exposure to sun irradiation, this was not a major contributory factor for vitamin D deficiency as a whole, in Saudi female students [35]. Supporting the results of Sedrani et al., no difference was reported in vitamin D level when compared among veiled and non-veiled Bangladeshi women [36]. Our present study supports the findings of Siddiqui and Hollick et al. showing vitamin D deficiency in girls due to limited sun light exposure.

A survey from Hong Kong showed that instead of having good knowledge about vitamin D, younger (middle-aged) women were ignorant of the role of sunlight in vitamin D production and expressed negative opinions regarding optimum exposure to sunlight [21]. In our study, the ignorance about basic concepts of vitamin D was higher in girls than boys, as evidenced by their significantly higher use of sunscreen (p < 0.001). This lack of knowledge became more severe, when we observed that the use of sunscreen in girls was surprisingly common, in spite of knowing and responding that they were fully covered during sun exposure (p < 0.02). So vitamin D deficiency in girls is higher as a result of the double negative effects of using sunscreen, as well as fully covering their body.

### Mean vitamin D levels and vitamin D knowledge

As is evidenced from Table 3, irrespectively of gender, the mean level of vitamin D was significantly different based only at the correct responses of different questions. For instance, the group of girls who were observed with significantly higher level of vitamin D were only those who responded correctly to the question related to disease and source of vitamin D (p = 0.03, and 0.015, respectively). Similarly, the mean vitamin D levels was significantly higher in both boys and girls responding “yes” to the question “Have you heard about vitamin D sources in primary care centers” as compared to “No” (p-value 0.03, and 0.05, respectively).

## Conclusions

High prevalence of vitamin D deficiency in both genders especially girls was observed in this study. The main possible reasons for this deficiency was the lack of knowledge about vitamin D (lack of sun exposure, use of sunscreen and fully covering the body during sun exposure), mostly among girls. Schools and primary care centers were not able to provide information on the importance of vitamin D in bone health, growth and development of children. Awareness of the benefits of sunlight needs to be increased by provision of specific about how often sun exposure is required, the duration, and how much of the body should be exposed for optimal vitamin D uptake. Physicians must have more nutritional knowledge and health care information about vitamin D that could be transferred easily to children’s. Creating more areas where women can uncover freely may have to be part of the strategy of preventing vitamin D deficiency. In girls, increasing incidental sun exposure through routine, daily, outdoor activities will help increase sun exposure for vitamin D activation. Consequently, further efforts to educate children about importance and role of vitamin D through sources like media and health professionals are needed in near future.

## Abbreviations

BMI: Body mass index
DEQAS: vitamin D External Quality Assessment Scheme
KSA: Kingdom of Saudi Arabia
MOH: Ministry of Health
VDR: vitamin D receptor
